# Supplementary material for: Complex‐centric proteome profiling by SEC‐SWATH‐MS
Source: Mol Syst Biol. 2019 Jan 14;15(1):e8438. doi: 10.15252/msb.20188438 (PMC6346213; doi:10.15252/msb.20188438)
Supplement: Supplementary file 8 — Dataset EV7 [file MSB-15-e8438-s008.zip › feature_plots_string/P51149.pdf]

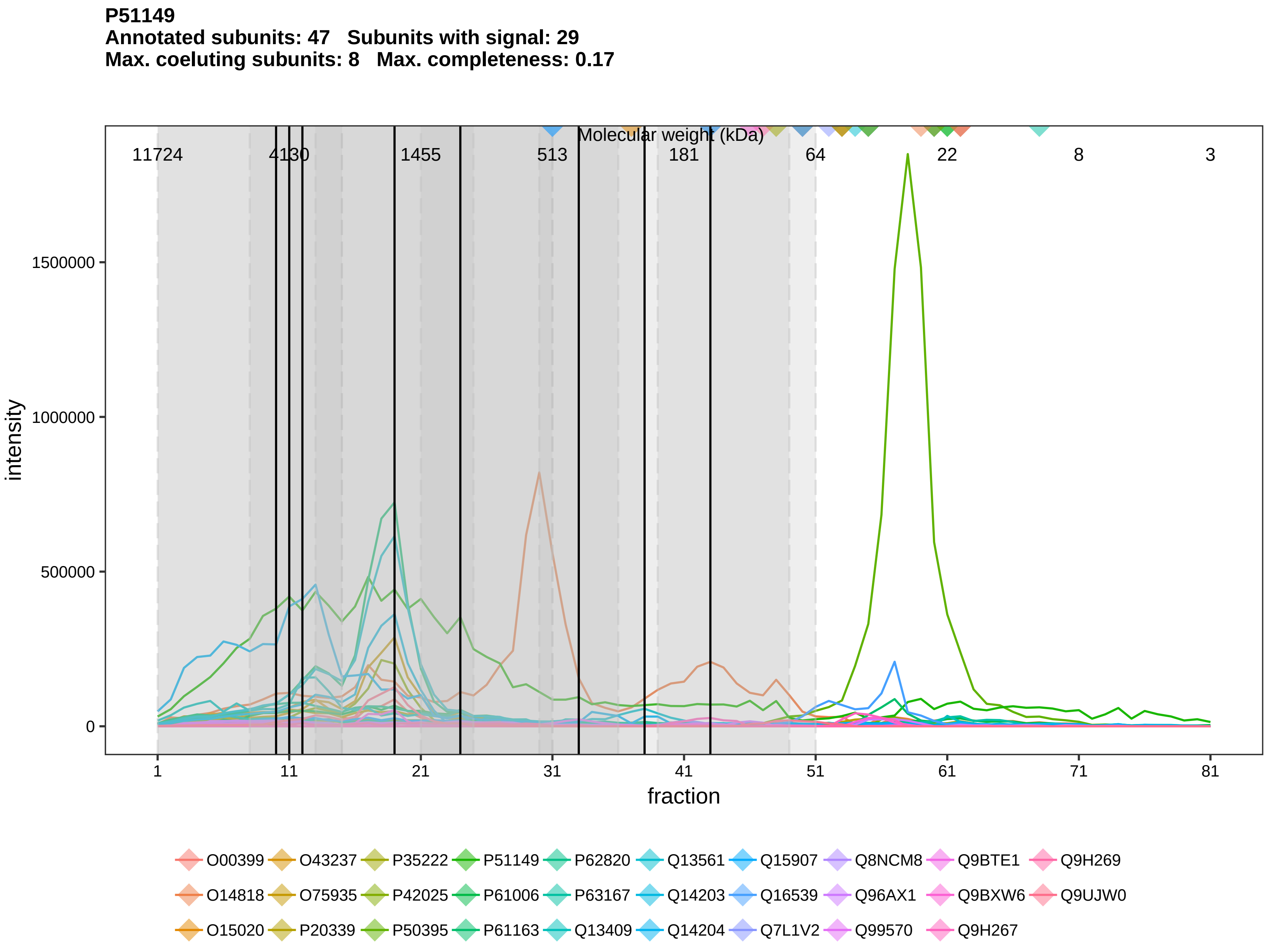

P51149
Annotated subunits: 47 Subunits with signal: 29
Max. coeluting subunits: 8 Max. completeness: 0.17
Molecular weight (kDa)
11724
4130
1455
513
181
64
22
8
3
intensity
1500000
1000000
500000
0
1
11
21
31
41
51
61
71
81
fraction
O00399
O43237
P35222
P51149
P62820
Q13561
Q15907
Q8NCM8
Q9BTE1
Q9H269
O14818
O75935
P42025
P61006
P63167
Q14203
Q16539
Q96AX1
Q9BXW6
Q9UJW0
O15020
P20339
P50395
P61163
Q13409
Q14204
Q7L1V2
Q99570
Q9H267
